# Antibiotic Resistance in *Escherichia coli* from Broiler Chickens After Amoxicillin Treatment in an Experimental Environment

**DOI:** 10.1089/mdr.2019.0442

**Published:** 2020-09-09

**Authors:** Elke Burow, Mirjam Grobbel, Bernd-Alois Tenhagen, Céline Simoneit, István Szabó, Daniela Wendt, Corinna Kürbis, Mechthild Ladwig-Wiegard, Stefanie Banneke, Annemarie Käsbohrer

**Affiliations:** ^1^ Department Biological Safety, German Federal Institute for Risk Assessment, Berlin, Germany.; ^2^ Department Safety in the Food Chain, German Federal Institute for Risk Assessment, Berlin, Germany.; ^3^ Department Experimental Toxicology and ZEBET, German Federal Institute for Risk Assessment, Berlin, Germany.; ^4^ Department for Farm Animals and Veterinary Public Health, Institute of Food Safety, Food Technology and Veterinary Public Health, University of Veterinary Medicine Vienna, Vienna, Austria.

**Keywords:** antimicrobial, chicken, cephalosporin, spread, susceptibility

## Abstract

Groupwise antibiotic treatments are common in broiler chicken production. They induce selection for antibiotic resistance in commensal *Escherichia coli.* This study aimed to investigate antibiotic resistance after individual (I, drenching) or groupwise treatment (G, by water) with amoxicillin, and after contact with I or G (KI or KG), compared with untreated broilers without contact with treated broilers (C), and pretreatment values. Finally, we compared antibiotic resistance from broilers (G) after a second treatment, with a treatment in the contact animals (KG), and a first treatment in the control animals (C). Resistance to ampicillin and other antibiotics was significantly increased in groups G and I within 2 days, suggesting (co-)selection of resistance. The increase was lower in groups KI, KG, and C during the first treatment (days 1–5). The increased resistance in group C was interpreted as a change in the microbiota after initial moving and first feeding. After treatment, resistance rates decreased to initial or lower values in all groups. During the second treatment period (days 34–38), all three groups' (G, KG, and C) resistance levels increased to equally high levels. Cephalosporin resistance was low, and did not change over the experimental period. On days 3 and 38, resistance rates of *E. coli* from duodenum, jejunum, and cecum did not differ between segments and treatment routes. Overall, the baseline levels of antibiotic resistance in *E. coli* were high. Amoxicillin triggered an increase in resistance levels, irrespective of the mode of treatment. Substantial resistance dynamics in untreated controls warrant further investigation.

## Introduction

The production of chicken meat (109 million tons) constituted 33% of the total global meat production in 2017.^1^ According to German surveillance data (first half of 2019), broiler chickens are among the top users of antibiotics in animal production, second only to turkeys.^2^ In Germany, penicillins (including amoxicillin) constituted 37% of the weight of all antibiotics sold for the use in veterinary medicine in 2017,^3^ and are the third most commonly used antibiotic in broilers.^4^ Antibiotic treatment has been found to be associated with an increase in resistance frequency in *Escherichia coli* in chickens across various studies.^5–7^ During and after amoxicillin treatment, the resistance rate to amoxicillin was 50–100%.^8–11^ Commensal *E. coli* from animals can serve as a reservoir for antibiotic resistance genes relevant for human commensal^12^ and pathogenic^13^ bacteria. In the national monitoring on zoonotic and commensal bacteria, *E. coli* from broilers are most frequently resistant to aminopenicillins (60–80% to ampicillin since 2011).^14^ Antibiotic-resistant bacteria can be transferred along the food chain.^15^ Penicillins and cephalosporines are highly and critically important antibiotics in veterinary and human medicine.^16,17^ Preservation of the effectiveness of these and other antibiotics is a global target.^18^

Group treatments are common in treating infections in broiler production systems. Most antibiotic drugs for chickens are currently approved for oral administration.^19^ Therefore, antibiotics are offered to all group members, including sick and also healthy animals.^20^ The amount of drug intake differs between the individuals in a group treatment.^21^ In theory, individual oral treatment could address only sick broilers, and guarantee for exact intake. This reduces the number of exposed animals, assures proper dosing, and might therefore reduce selection pressure, development, and spread of resistance in the group. However, experimental studies^22,23^ suggested that residual antibiotics and resistant bacteria can be transferred from treated to untreated animals housed in the same building, or moved through the same barn sections. Little is known about the potential risk for increased resistance in bacteria from untreated chickens in direct contact with treated animals.

Furthermore, there is limited understanding of the possible effects of repeated antibiotic treatments on antibiotic resistance occurrence, and of resistance occurrence, to antibiotic agents other than beta-lactams after amoxicillin treatment in chickens. Resistance of *E. coli* to aminoglycosides and chloramphenicol, in addition to beta-lactams after repeated oral treatment with amoxicillin, has been described for broilers.^24^

After oral administration of amoxicillin to broilers, amoxicillin is resorbed almost completely from the gastrointestinal tract.^19^ Therefore, a concentration gradient in the intestinal tract can be expected, with the concentration in the distal parts of the intestine (colon) being lower than in the more proximal (jejunum) ones. This has been observed for enrofloxacin in pigs.^25^ Whether this gradient per intestine section has an impact on the development of resistance is an area that requires further study. A previous study showed differences in the proportion of resistant bacteria between the cecum and the excreted feces.^26^ It also remains to be investigated whether there are differences between very young animals at the first treatment, and mature animals in the later treatment, and between first and second treatment.

Our aim was to evaluate the effect of individual and groupwise oral administration of amoxicillin on the occurrence of resistance to ampicillin and other antibiotics in commensal *E. coli* from treated and untreated contact broilers during their life. A second aim was to study the effect of repeated treatment on antibiotic resistance. Our hypotheses were as follows: (1) *E. coli* present in the gut of broiler chickens would show a temporary increase in resistance to penicillins and other antibiotic agents after early treatment with amoxicillin, compared with pretreatment and untreated controls; (2) the increase would differ between individually and groupwise-treated animals, and their respective contact animals; (3) during a second treatment at the end of the fattening period, resistance would be higher in previously treated animals than in animals treated for the first time; and (4) resistance occurrence would differ between *E. coli* from different intestinal segments.

## Materials and Methods

### Animals and study groups

**FIG. 1.**
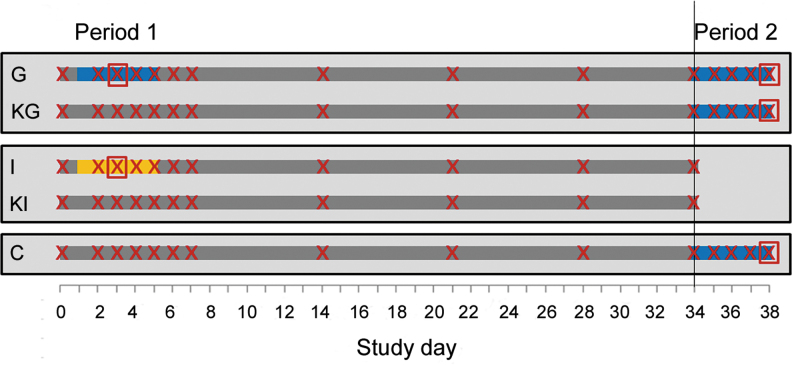
Experimental design: study period, treatments (*blue bar* = groupwise administration of amoxicillin through water, *orange bar* = individual administration of amoxicillin through drencher) and cloacal swab sampling (*crosses*) as well as blood sampling and intestinal content sampling (*rectangles* around *crosses*) per study group (C, control; G, treated groupwise; I, treated individually; KG, contact to G; KI, contact to I), *rectangles* with *black framing* indicate groups housed in one room.

We obtained 125 five-day-old clinically healthy chicks of Ross 308 from a single hatchery (Supplementary Data). Each of the 125 chicks was assigned to 1 of the 5 groups: I, individual treatment with amoxicillin (through drencher); G, groupwise treatment with amoxicillin (through drinker); KI, contact with I; KG, contact with G; or C, control that was treated late in the study (Fig. 1). The chicks were randomly allocated into these five groups of 23 animals per group, by picking one chick after the other out of the breeder's transport box and placing every jth chick into groups I, KI, G, KG, and C. In picking turns 2, 7, 12, 17, and 22, one additional chick was placed into groups G and I for early blood sampling. The sample size calculation was based on 76.8% risk after treatment, compared with 14% risk before or without treatment, 95% confidence interval, 80% power, and two-sided testing. Four animals were added per group to account for potential loss. All broilers were vaccinated against infectious bronchitis, and Gumboro and Newcastle diseases.

### Housing

The study groups were housed in three separate rooms: Groups G and KG were housed together, as were groups I and KI, whereas group C was housed alone. The rooms were accessed from the same corridor but a door separated the part of the corridor leading to the control room. During the first 5 study days (antibiotic treatment), groups G and KG, and I and KI, were separated by a 30 cm high fence to allow application of the drug by drinking water to G, without providing group KG with access to the drug. In groups I and KI, the same construction was chosen to ensure for better comparability. The small fence enabled broilers to still have physical contact and to breathe the same air in the room. Litter, including spoiled bedding, was exchanged between the treated and contact groups. We exchanged roughly 50% of the litter between the groups to align the environmental conditions between both groups. This was carried out to ensure that the only difference between the treated and contact animals would be the treatment. After 5 days, the fence was removed (see Supplementary Data for additional information on housing and feeding).

### Study period

The trial was carried out from January to February 2015. After assignment to the groups, the chicks had 2 days to get settled and to get accustomed to the facility and the caretakers. After these first 2 days of life, the study started and the first study period ran for 34 days in all groups. The broilers in groups I and KI were killed on day 34 of the study. The broilers in groups G, KG, and C were kept until day 38 (second study period; Fig. 1).

### Antibiotic treatment

In the first treatment period (study days 1–5), groups I and G were treated with amoxicillin (20 mg amoxicillin per kg live weight and day) for 5 consecutive days (Fig. 1). In group G, amoxicillin solution was administered with the bell drinkers. The drug Octacillin^®^ (800 mg/g; Firma Albrecht GmbH) was dissolved in water, once in the morning and once in the evening with an interval of 12 hours (following application instructions). Animals in group I were treated with a curved application cannula in the morning. The dosage depended on each individual chick's live weight in group I. In group G, the dosage depended on the live weight of the group, and additionally on the group's water consumption. Dosage was adjusted daily based on the bodyweight and water consumption, as determined on the previous day (Supplementary Data).

In the second treatment period (study days 34–38), groups G, KG, and C were treated with amoxicillin through drinkers using the same method as for G in the first treatment period (Fig. 1). For group G, this was the second treatment. For group KG, this was the first treatment, but the animals had been exposed to the resistant bacteria, and presumably to drug residues beforehand. Group C served as a control group that was treated for the first time, and had not been exposed to the bacteria from groups G and KG before. There was no untreated control in this second treatment period.

The order of handling animals, biosecurity measures, and study rules are described in the Supplementary Data.

### Institutional and national guidelines for the care and use of laboratory animals

The study was approved by legal authorities of the Regional Office for Health and Social Affairs Berlin (LAGeSo, G 0175/14) according to the EU directive 2010/63/EU. The institutional officer for animal welfare reviewed the experimental study and all applicable institutional and national guidelines for the care and use of animals were followed. Experimental treatments of animals were classified as to lead to no worse than minor discomfort in the animals (daily health check, see Supplementary Data), owing to low pain of a very short duration (*e.g*., handling, application of a cannula for individual oral treatments, and repeated cloacal swabbing).

### Fecal sampling

#### Meconium samples

The inlets of the two transport boxes of chicks were cut into pieces and transferred into sterile blending bags containing Luria Bertani (LB) broth (Merck, Darmstadt, Germany) with 20% glycerol (Carl Roth GmbH). After 60 seconds of blending at highest speed, 5 mL per sample were stored at −80°C until further processing to investigate the growth of *E. coli* and their antibiotic resistance.

#### Cloacal swabs

Cloacal swabs were taken from all broilers on study days 0 (as reference), 2 to 7, 14, 21, 28, and 34, and from all broilers in groups G, KG, and C from days 35 to 38 (Fig. 1). The sampling on days 2–5 was carried out before the daily treatment. Within 1 hour after sampling, the swabs were transferred into cryo-tubes containing LB broth (Merck, Darmstadt, Germany) with 20% glycerol (Carl Roth GmbH), and stored at −80°C until further processing.

### Blood sampling

Blood serum was sampled from five broilers in groups I and G on study day 3, and from all broilers in groups G, KG, and C on study day 38 (Fig. 1), ∼1.5 hours after amoxicillin administration. The birds were electrically stunned, decapitated, and bled to death while the blood was collected into monovettes. On day 3, all blood was collected from the broilers of groups I and G, and blood collection from the broilers of groups G, KG, and C took place on day 38; blood samples of 5 mL were taken in all cases. The samples were centrifuged at 1,500 *g* for 10–15 minutes at room temperature. Serum was stored at −20°C until further processing.

### Intestinal content

Fecal content was collected from the intestines of the five birds killed for blood sampling in groups I and G on day 3, and of the five birds from groups G, KG, and C killed on study day 38 (Fig. 1). Samples from the intestinal sections duodenum, jejunum, and cecum were separated. Within 1 hour after sampling, the samples were stored in broth containing 20% glycerol and at −80°C until further processing.

### Detection of amoxicillin in blood serum

For the detection of amoxicillin, 200 μL of blood serum was spiked with 10 μL amoxicillin-d4 (50 μg/L serum) as internal standard, filled up to 1 mL with water/acetonitrile (90:10, vol/vol), vortexed, sonicated, and filtered using a 0.45 μm Phenex-RC membrane filter. The filtrates were directly analyzed using an Agilent 1260 Infinity LC coupled to a Sciex QTRAP-6500 system. Chromatographic separation was performed by injecting a 10 μL filtrate onto a Thermo Hypersil Gold column (150 × 2.1 mm, 3 μm). Within 10 minutes, the analysis was complete using the positive Electrospray ionization mode and the following transitions of multiple reaction monitoring: amoxicillin m/z 366 → 208 and m/z 366 → 349, amoxicilloic acid m/z 384 → 323 and m/z 384 → 367, amoxicillin diketopiperazine m/z 366 → 114 and m/z 366 → 160, amoxicillin-d4 m/z 370 → 212, and m/z 370 → 353. Method validation, which was carried out according to Commission Decision 2002/657/EC, showed linearity in the range of 2.5–80 μg/L, good reproducibility (<26%), and mean recoveries (103–116%) of 10 μg/L. The decision limits CCα, and the detection capabilities, were within the range of 5.3–6.2 and 7.8–11.7 μg/L.

### Isolation of *E. coli* and susceptibility testing

From each fecal sample, 100 μL were cultured overnight at 37°C on MacConkey agar (McC; Merck, DE), and McC with addition of 1 μg/mL cefotaxime sodium salt (McC+CTX; Merck). In parallel, nonspecific enrichment was performed in buffered peptone water (1:9, incubated overnight at 37°C) for all swabs and the meconium samples. If no *E. coli* grew on the primary plates, 100 μL of the enrichment broth was plated on McC to increase the detection rate.

One colony per sample with typical *E. coli* morphology was picked, identified as *E. coli* using Matrix Assisted Laser Desorption Ionization - Time of Flight Mass Spectrometry (Microflex Biotyper; Bruker), and preserved at −80°C. From McC+CTX, for every morphology present, one colony was chosen, and these colonies were then identified, and preserved at −80°C.

Determination of the minimum inhibitory concentration (MIC) was performed and validated as prescribed by the European Commission Implementing Decision No. 2013/652/EU^27^ on the monitoring and reporting of antimicrobial resistance in zoonotic and commensal bacteria, using commercial test plates (Sensititre; TREK Diagnostic Systems) containing 14 antimicrobial agents. Ampicillin resistance (MIC >8 μg/mL) was used as an indication of aminopenicillin resistance. Resistance to cefotaxime (MIC >0.25 μg/mL) or ceftazidime (MIC >0.5 μg/mL) was used as an indication of cephalosporin resistance. As no cutoff was available for azithromycin, a tentative cutoff was taken from data published by the European Food Safety Authority (EFSA).^28^

### Statistical analyses

The statistical analyses were carried out using SAS version 9.4 (SAS Institute, Inc., Cary, NC).

The detection of amoxicillin and/or its metabolites was dichotomized, as for the purposes of our study it only seemed relevant whether amoxicillin could be detected in the blood.

The probability of *E. coli* resistance to antibiotic agents (dichotomous outcome) was compared across study days and treatments, and across intestinal sections. All analyses were carried out using a logistic regression model (GENMOD procedure). The null hypothesis that the slope is not significantly different from zero was tested with *p* < 0.05 as the threshold for significance.

The analysis of body weight is described in the Supplementary Data.

## Results

### Animal losses and weight gain

During the study period, nine broilers (two in each of groups KG, I, C, and three in group G) died or were killed. Each study group was represented by at least 20 broilers until the end of the study period. Samples collected from killed or euthanized animals before their death were included in the study. Several broilers (mainly in group G) showed decreasing ability to walk at the end of the study period. The weight gain during the study period was similar for all groups and there was no significant difference between the weights in the groups on study day 38 (Supplementary Data).

### Intake of medicated water

The water consumption during treatment through drinkers did not differ between groups (Supplementary Data).

### Amoxicillin in blood serum

#### First treatment period

Amoxicillin, amoxicillin acid, or amoxicillin diketopiperazine were detected in the blood serum in two of the five broilers from group I and in all five sampled broilers from group G on study day 3 (third day of treatment).

#### Second treatment period

After the second treatment period, that is, on study day 38 (fifth treatment day), the detection rates for amoxicillin and its metabolics in groups G, KG, and C were 65%, 62%, and 100% and lower than during the first group treatment period.

### Antibiotic resistance in *E. coli*

#### Meconium samples

The two tested isolates from meconium samples were sensitive to all 14 antibiotic agents. The lowest possible MIC was observed for 12 antibiotics while ampicillin and azithromycin displayed third and second lowest concentration. Selective cultivation of *E. coli* from the meconium on McC+CTX after nonspecified enrichment showed no growth.

#### Cloacal swabs

**FIG. 2.**
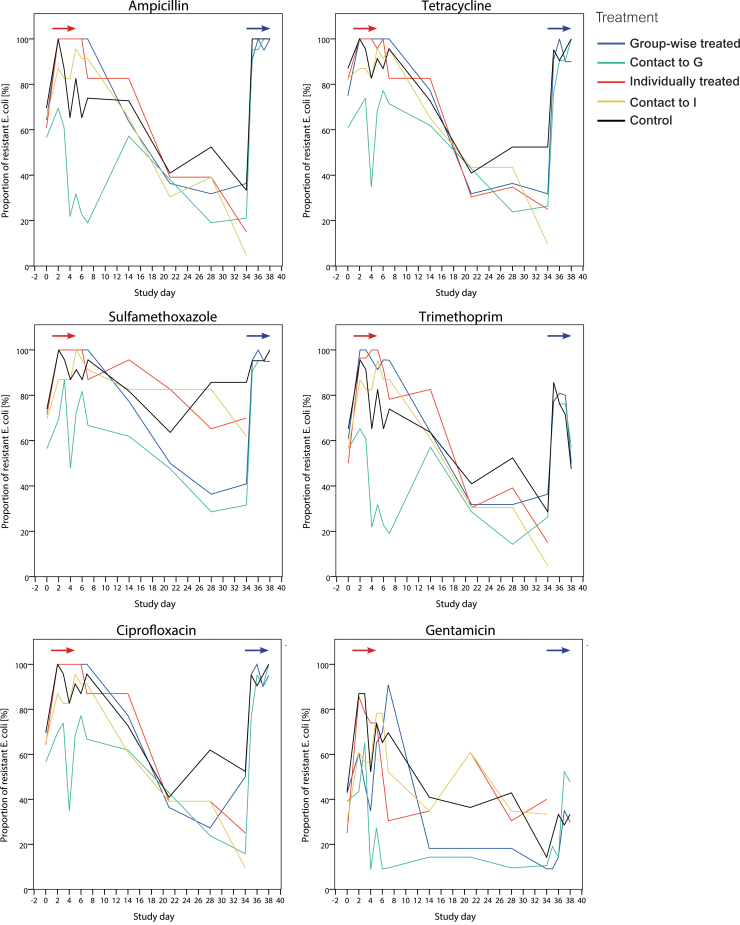

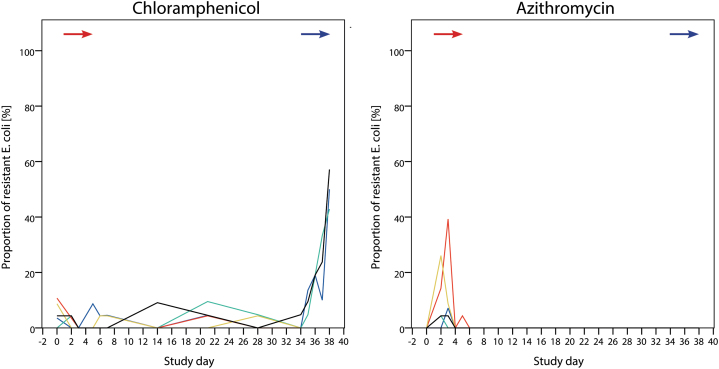
Proportion of *Escherichia coli* from cloacal swabs of broilers that were microbiologically resistant to the respective antibiotic agents after amoxicillin treatment applied on days 1–5 and on days 34–38. The *red arrow* indicates the period of treatment in broilers treated individually and groupwise (days 1–5). The *blue arrow* indicates the period of groupwise amoxicillin treatment in the groups G, KG, and C (days 35–38).

*Escherichia coli* isolates were obtained from cloacal swabs per bird and sampling day, leading to a total 1,516 isolates—at least 20 per study group and day. Proportions of isolates with antibiotic resistance per agent, treatment, and sampling day are presented in Fig. 2. Patterns of resistance rates of the quinolones nalidixic acid and ciprofloxacin were identical (only ciprofloxacin presented). No isolate tested was resistant to cefotaxime and <20% of the isolates were resistant to ceftazidime (cephalosporines), tigecycline, colistin, and meropenem at single days without any significant increase/decrease (data not shown).

Initial resistance levels were high and similar in all study groups, including the control, with regard to ampicillin, tetracycline, sulfamethoxazole, trimethoprim, ciprofloxacin, and nalidixic acid (50–90%). The frequency of resistance to these agents increased significantly (*p* < 0.0001 to *p* < 0.05 per agent and day) in the treated groups from the initial sampling to the second study day (reached 100%) and following days (first treatment period). A significant (*p* < 0.05) increase was also found in the contact groups and control, although on a significantly lower level compared with the treated groups. The control group had, starting with day 0, similar levels of resistance to agents, as seen in the treated and contact groups. Group KG generally showed the lowest frequencies of resistance to most agents, initially and until the second treatment period. In groups G, KG, and C, resistance rates decreased slightly on day 4 and showed a second peak for most agents at day 5. After the cessation of the first amoxicillin treatment (day 7), proportions of resistance to most antibiotic agents decreased and reached initial or even lower levels in most study groups between study days 28 and 34. For ampicillin, this decrease led to a significantly lower level in groups G (*p* = 0.0214) and KG (*p* = 0.0093) on day 28 compared with day 0.

In the second treatment period (including control), the resistance rates for most antibiotic agents increased rapidly from study day 34 (onset of second treatment) to day 35, and reached 100% in all the now groupwise-treated G, KG and C groups. This course was uniform across study groups, and resistance levels for ampicillin, tetracycline, sulfamethoxazole, ciprofloxacin, and nalidixic acid persisted until the end of the study.

The pattern of gentamicin resistance was slightly different from the patterns of most other agents. Initially, ∼40% of the tested *E. coli* were resistant to this agent in each group. Proportions increased earlier in groups I and KI compared with groups G and KG, and in the second treatment period, the increase was less homogenous between groups than it was for other agents. Increase in resistance to azithromycin in groups I and KI during first treatment period and to chloramphenicol in groups G, KG, and C during second treatment period occurred at a low but significant level. In the second treatment period, the proportions of resistance to chloramphenicol in group G were significantly higher compared with the first treatment period, but lower than or similar to in groups KG and C.

### Antibiotic resistance in *E. coli* from intestinal segments

On study and treatment day 3, all sampled *E. coli* from the three intestinal sections were resistant to the antibiotic agents ampicillin, tetracycline, ciprofloxacin, and nalidixic acid in the treated groups I and G. None of the isolates were resistant to cefotaxime and ceftazidime. Resistance to trimethoprim was present in 80–100% and to gentamicin in 40–80% of the isolates in both groups and intestinal sections.

**FIG. 3.**
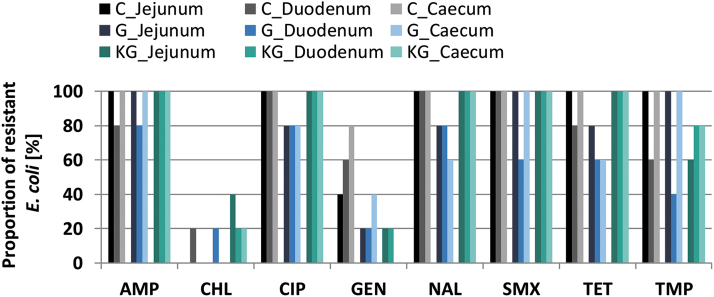
Proportion of resistant *Escherichia coli* from broiler's intestine (five broilers per group) on study day 38 (C, control; G, treated groupwise; KG, untreated contact to G). AMP, ampicillin; CHL, chloramphenicol; CIP, ciprofloxacin; GEN, gentamicin; NAL, nalidixic acid; SMX, sulfamethoxazole; TET, tetracycline; TMP, trimethoprim.

At the end of the second treatment period (day 38), the *E. coli* from groups G, KG, and C showed resistance to the same antibiotic agents as in groups I and G on study day 3 but proportions were lower for several intestinal sections and treatments (Fig. 3). In addition, up to 40% of the *E. coli* from each group were resistant to chloramphenicol.

There were no significant differences between the probabilities of an *E. coli* isolate to being resistant in the intestinal sections of the respective study groups at days 3 or 38. Comparing group G across both days, the antibiotic resistance rates of the intestinal sections did not significantly differ for most agents, except for trimethoprim (*p* = 0.0192) and gentamicin (*p* = 0.0496) resistance in the duodenum, which was significantly lower on day 38 compared with day 3.

## Discussion

### Antibiotic resistance in *E. coli* from cloacal swabs

Our study investigated the occurrence of antibiotic resistance in commensal *E. coli* from broiler chickens after they had been exposed to individual or groupwise treatment, or had been in contact with the treated broilers. Therefore, we evaluated isolates from broilers in the respective experimental units at several time points before, during, and after administration of antibiotics and during a second treatment period.

### Increase of resistance in treated broilers

*E. coli* from broiler chickens showed a temporary increase in resistance to ampicillin and other antibiotic agents (tetracycline, sulfamethoxazole, trimethoprim, ciprofloxacin, nalidixic acid, gentamicin, azithromycin, and chloramphenicol) after the early treatment compared with pretreatment. This finding confirmed the respective part of hypothesis 1.

The observed increase in ampicillin resistance in broilers after amoxicillin treatment was in accordance with findings in previous studies.^11,24^ Resistance rates to agents from other antibiotic classes (tetracyclines, aminoglycosides, sulfonamides, quinolones, and macrolides) also increased. Similarly, Jiménez-Belenguer *et al.*^24^ found increased resistance to tetracycline, aminoglycosides, and chloramphenicol after amoxicillin treatment. These findings suggest coselection for resistance to these antibiotics.

It was considered likely that amoxicillin treatment would also cause a (temporary) increase of resistance to cephalosporines (cefotaxime and ceftazidime), but this was not observed. Jiménez-Belenguer *et al.*^24^ also did not find any increase in resistance to ceftriaxone (third-generation cephalosporin, like cefotaxime and ceftazidime) at the end of broiler chickens' life after repeated amoxicillin treatment. In contrast, Furtula *et al.*^29^ detected 45% resistance to ceftiofur (third-generation cephalosporin) after amoxicillin treatment in chickens. Besides, we froze samples after collection. Freezing of samples is known to lower the bacterial viability and might have had an impact on the total number of viable cells in the sample. Therefore, resistant bacteria that were in the samples in very low amounts might have been undetected. However, our results suggest that there were no genes conferring resistance to cefotaxime in the study population and these genes also did not arise during or after treatment.

With regard to the unexpected increase in resistance in the control group, which was, however, significantly lower than in the treated groups, we can rule out treatment of controls. Transmission of bacteria from the treated to the control group was highly unlikely because of the hygienic barriers between the groups (Supplementary Data). The early increase in resistance proportions in the controls underlines the rapid evolution of the intestinal microbiota during the first days of life in chickens.^30^

### Difference in resistance rates between treated and contact groups

The increase in resistance rates did not significantly differ between broilers treated individually or groupwise (rejection of the respective part in hypothesis 2). Resistance rates in the contact groups increased significantly while the treated groups received amoxicillin, but the increase was significantly lower in the contact group compared with the treated broilers (confirmed hypothesis 2).

In both the groups treated individually and the one treated groupwise, all broiler chickens were treated to study the effect of the administration route itself. It is an open question whether individual treatment of only few broilers per group would have the potential to reduce resistance rates. Previous on-farm observations of pigs found lower risk for resistance associated with individual and parenteral treatment compared with groupwise treatment.^31,32^

High baseline levels of resistance may have prevented significant differences between the treated groups and the contact groups from being detected. Because treated groups actually showed significantly higher and more persistent resistance rates compared with the contact groups, direct exposure to antibiotics was probably a stronger trigger.

Because resistance levels in the control were high, we cannot clearly distinguish between contact effects and the evolution of resistance in the contact group. However, the significant increase in resistance rates in the contact broilers may, to some extent, be owing to the intake of drug residues that were shed by the treated animals. This has previously been described for untreated pigs.^22^ Furthermore, it is likely that bacteria were exchanged between treated and untreated animals housed and moved in the same environment. Wiuff *et al.*^23^ found that untreated pigs carried enrofloxacin-resistant *E. coli* after they were kept in an environment where enrofloxacin-treated pigs had been before. The contact broiler chickens in this study were exposed to treated broiler chickens' feces. Therefore, it was likely that they ingested resistant bacteria. A study on laying hens found chickens being colonized with *Salmonella* after intracloacal inoculation.^33^ Hence, intrusion of bacteria into the cloaca with subsequent colonization of the chicken gut might also be possible but is likely to play a minor role.

### Antibiotic resistance after repeated treatment

During the second treatment period, resistance rates increased rapidly to up to 100% in all three treated groups. This suggests that the resistant strains were still prevalent in the population and under selection pressure quickly outcompeeted the susceptible strains. By contrast, Jiménez-Belenguer *et al.*^24^ detected a slightly decreased frequency of resistance to ampicillin and amoxicillin after double amoxicillin treatment at day 21 compared with after the first treatment at day 7 of life in broilers. Overall, our study did not confirm higher resistance rates in pretreated broilers compared with broilers treated for the first time (hypothesis 3 rejected).

### Antibiotic resistance in *E. coli* from intestinal content

We compared occurrence of antibiotic resistance in *E. coli* sampled from the different intestinal segments of broilers. It turned out that there was no difference between segments in the first and the second treatment. Likewise, resistance levels in *E. coli* from intestinal contents did not differ between the early and the late treatment in group G. Hence, hypothesis 4 was rejected. Differences between the intestinal segments had been expected as drug concentrations may differ along the intestinal tract owing to absorption of the drug from the jejunum. These differences might even increase as the broilers grow (see also Supplementary Data) and the intestine becomes longer. In pigs, a lower concentration of enrofloxacin was detected in the colon compared with the jejunum.^25^ Another study showed more consistently increased MIC values for *E. coli* from the cecum compared with those from cloacal feces in 15-day-old chickens.^26^ Apart from these studies, Moro *et al.*^34^ found resistant *E. coli* move with motility and peristalsis from the upper to the lower part of the intestine in swine. The broilers' short intestinal tract with short feed passage times may have contributed to the homogenous picture of resistant *E. coli* along the intestine on study days 3 and 38 in this study. As the resistance levels in the colon were similar in groups G and I, the shedding of resistant *E. coli* and exposure of contact animals may have been similar for groups G/KG and I/KI. Method of administration therefore is not likely to affect excretion of resistant bacteria when using the same dose of amoxicillin.

### Antibiotic resistance before treatment

Individual cloacal samples from the second day of life (study day 0), before the first treatment day, showed high resistance levels to several agents in all groups. The animals' intestines were seemingly already colonized with resistant bacteria before the first treatment. The rapid increase in resistance during treatment is probably based on the multiplication of these resistant bacteria. The experimental rooms had been checked for the presence of Enterobacteriaceae before the arrival of the chicks and the transport boxes had not been used before (Supplementary Data). The bedding material (softwood, see Supplementary Data) cannot be ruled out as a potential source of bacteria as it was not tested or treated for the absence of Enterobacteriaceae. Wood shavings were earlier identified as a potential reservoir for transmitting multidrug resistance, including ceftriaxone resistance.^35^

In the German monitoring of antimicrobial resistance, 26.3–27.3% of commensal *E. coli* from chicken breeding herds showed resistance to ampicillin in 2013 and 2014.^36,37^ This is a more probable source of the observed early colonization with resistant *E. coli*. Contamination of day-old chicks with bacteria originating from the breeding herd or the hatchery environment has previously been described with respect to Extended Spectrum Beta-Lactamase forming *E. coli*.^38,39^

In the course of the experimental period, the proportions of resistant *E. coli* returned to initial or even lower levels in all groups including the control. This suggests that susceptible *E. coli* strains outcompeeted the resistant ones and that the continuously used bedding material was of minor relevance. Anyway, even with no antibiotics administered to broilers, their established microbiome was already a reservoir for antibiotic-resistant *E. coli*.

### Exposure to dose

The antibiotic dose offered to the animals was identical for the different groups and water intake did not differ between them (Supplementary Data). Amoxicillin and its metabolics were, however, detected in fewer broiler chickens from group I compared with those from group G in the first treatment period. This finding is based on a small sample size, but it suggests that all sampled broilers in group G had taken up the medicated water and absorbed the drug while there was a less reliable blood concentration in broilers drenched once daily.

After the second treatment period, detection rates were lower in broilers from groups G and KG compared with broilers from group C. In groups G and KG, the broilers had drunken less, although not significantly less, than in group C. For group G, this can be explained with locomotion and general impairments in several broilers during the treatment period. After the death of two broilers from study days 35 to 37, the average amount of drunken water per bird increased in group G. In general, the broilers were limited in their ability to walk, and may have avoided moving to reach resources at the end of their life.

Consequently, the dose the individual broilers were exposed to during the treatment days may have differed between individual animals in group G because of the unstable individual intake of drinking water. In the literature, the association of doses with the occurrence of resistance is not clear in chickens and pigs. Therapeutic dosages led to a similar increase in resistance levels as subtherapeutic dosages.^7,40^ In our study, we cannot distinguish between the impact of route and dose as we did not measure the exact drug intake per individual in group G. Therefore, we did not consider dose in the analysis.

## Conclusions

Our study confirmed that amoxicillin treatment triggered an increase in resistance levels for several antibiotics for a limited period of time. We found no significant differences between individual and group treatments—as long as the same number of animals is treated. That means that group treatments through drinking water as such are not inferior to individual treatments with respect to the development of resistance. In line with that, we also did not find differences between the two contact groups. We did not find differences in the resistance levels of *E. coli* from different segments of the intestinal tract. Neither did we find a difference between animals that had been pretreated and those that were treated for the first time. However, we did find high initial levels of antibiotic resistance in chicks before treatment and substantial resistance dynamics also in untreated groups that warrant further investigation. Both may have had a substantial effect on our study results. Therefore, further studies challenging our results are required.

## Supplementary Material

Supplemental data
