# Supplementary material for: Antibiotic Resistance in Escherichia coli from Broiler Chickens After Amoxicillin Treatment in an Experimental Environment
Source: Microb Drug Resist. 2020 Sep 9;26(9):1098–107. doi: 10.1089/mdr.2019.0442 (PMC7482129; doi:10.1089/mdr.2019.0442)
Supplement: Supplemental data [file Supp_Data.pdf]

Supplementary Data

Supplementary Materials and Methods

Transport of animals

On their day of hatching, the chicks were transported in one-way perforated cardboard boxes with institutional, wet-cleaned and disinfected (Calgonit sterizid Kokzi PRO®, 1% cresol; Calvatis GmbH, Ladenburg, Germany) vehicles to the experimental facilities at the German Federal Institute for Risk Assessment in Berlin.

Housing

Each of the five groups had an area of 6.5 m^2 during the first 5 days, thereafter groups I and KI had a total of 13 m^2, with groups G and KG having the same. Each room was equipped with softwood bedding, air filtration, waste water disinfection, a separate hygiene lock, a water stop (no connection between rooms or hand washbasins in the hygiene lock), and separate feed supply.

Feeding

The chicks were fed rations in two stages. The change of food was after 3 weeks with several days of transition in between. The feed was supplied in troughs, and the water in bell drinkers for each group (both *ad libitum*).

Handling procedure

On sampling days, all handling was carried out on each chick separately in a separate room in the same hygienic unit to prevent as far as possible cross-contamination and contamination of the pen with drugs resulting from the handling procedure. Control/contact animals were handled before treated animals, to avoid cross contamination, for example, with drugs or bacteria through the handling procedure.

The broilers were handled in the morning before the water bells and feeding troughs were cleaned and refilled. Each broiler was manually restrained and submitted to its group-specific procedure before being returned to its pen.

Health and weight check

All broilers were visually checked for their general condition, feeding behavior, and body weight every morning throughout the study period. All broilers were weighed 1 day before the start of the study, every day from study days 1 to 7, then subsequently once a week until study day 28, and again daily from study days 34 to 38. The body condition and feeding behavior of the broilers was scored using a clinical examination protocol on study days 1–7.

Biosecurity measures and study rules

The animal rooms were cleaned, disinfected, and checked for presence of Enterobacteriaceae using dip slides (Roti-DipSlide VRBD; Carl Roth GmbH) on the floor, wall, door, ceiling, ventilation, feeder, drinker, barrier, and pipe before the chicks were enrolled.

While the broilers were being housed, the rooms were cleaned every morning. The floor was dry cleaned and the litter completely exchanged once a week, the drinkers were wet cleaned every day and the feeders when needed. In the daily routines, the groups were either cared for by different persons working in parallel, or by the same person in a given sequence visiting the control group first, then group I/KI, and finally group G/KG. Personnel were not allowed to go back from groups G/KG to I/KI, or to C the same day without showering and changing their clothes first. Each room system was entered wearing a new single-use overall (also covering the head), disposable gloves, a face mask, and disinfected rubber boots that remained in the respective hygiene sluice during the whole study period.

Broilers showing a minor disorder of welfare were treated within their group. Antimicrobial treatment, other than that required for the study, was not permitted. If necessary, the animal was separated within the room, remaining in contact with its group. Broilers that were seriously ill without prospect of recovery were removed from the trial and killed.

Statistical analysis

The mean body weight was compared across study groups. As the assumptions of normal distribution and variance homogeneity (on day 6) were partly violated, all comparisons were carried out with both analysis of variance (assumption of normal distribution, variance homogeneity; MIXED procedure) and nonparametric tests (assumption of at least ordinal distribution; Kruskal--Wallis for study group; npar1way procedure) to test the null hypotheses that mean values and mean ranks were equal between the study groups.

Supplementary Results

Body weight

The mean body weight (±standard deviation) among all broilers was 67.7 ± 5.9 g, $2{,}653.3 \pm 296.7$ g, and $3{,}177.6 \pm 353.0$ g on study days 1, 34, and 38. The mean body weight was significantly ($p < 0.05$) lower in group I compared with groups KG, KI, and C, significantly ($p < 0.05$) higher in group KG compared with all other groups on study day 1, and significantly ($p < 0.05$) lower in groups I and KI compared with group KG on study day 34, in the analysis of variance and accordingly in nonparametric tests.

Intake of medicated water

Calculated from rest water in the drinkers, a chick in group G drank on average 65 mL on each of the first three treatment days (study days 1–3), and 90 and 95 mL on days 4 and 5, when the group size was reduced from 28 to 23 chicks (5 chicks killed for blood sampling). During the last four study and treatment days, a broiler in groups G, KG, and C drank on average 476, 506, and 526 mL, respectively (no significant differences between groups), and during the last day before killing, on average 365, 329, and 405 mL.
